# A case of patellar fractures in monozygotic twin gymnasts

**DOI:** 10.1186/1758-2555-4-20

**Published:** 2012-06-12

**Authors:** Andrew J Beamish, Gareth L Roberts, Peter Cnudde

**Affiliations:** 1 Department of Trauma and Orthopaedic Surgery, West Wales General Hospital, Hywel Dda NHS Trust, Dolgwili Road, Carmarthen, Wales, SA31 2AF, UK

**Keywords:** Patellar fracture, Twins, Gymnast, Bone mineral density, Genetic

## Abstract

We present a case of near identical patellar fractures in adolescent monozygotic twins who are both high-level competitive gymnasts. These patients presented 14 months apart with almost identical history and clinical findings. Both had an intense training regime involving over 30 hours per week of load-bearing exercise. Clinical and radiological examinations suggested avulsion or sleeve fracture of the inferior pole of the patella with minimal displacement. Diagnoses of patellar stress fracture with avulsion of the distal pole and symptomatic bipartite patella could not be reliably excluded. Both fractures were treated conservatively with immobilisation of the knee in extension. An excellent functional result was observed in both patients with return to full activity at 8 weeks.

This is the first published case of identical injury to the patella in monozygotic twins. A significant genetic influence on bone mineral density (BMD) has been reported and low BMD is associated with increased susceptibility to fracture. These injuries corroborate a genetic influence on susceptibility to fracture. There is a requirement for further work to investigate genetic factors influencing susceptibility to fracture.

## Background

The patella is the largest sesamoid bone in the body. It is formed within the tendon of the quadriceps femoris muscle as it crosses anterior to the knee joint. Approximately 1% of all skeletal injuries in the adult and child are patella fractures [1-3]. Transverse fractures, including avulsion fractures, commonly result from indirect trauma, especially in the young [1]. A diastasis of 3 mm between fragments is diagnostic of a displaced fracture [1,4].

We report two near identical patella cases of fracture in adolescent, male identical twins, both competitive gymnasts aiming for the 2012 Olympic Games. Both had an intense training regime involving over 30 hours per week of load-bearing exercise. This regime included running, jumping and landing from height. Both fractures were treated conservatively with immobilisation of the knee in extension. An excellent functional result was observed in both patients.

## Case Presentation

### Case 1

A healthy 13-year-old male presented to the Emergency Department with acute onset of anterior left knee pain. This occurred during a gymnastics training session as he took off for a tumble. He described hearing a “crunch” sound as he extended the left knee upon take-off. Prior to this injury he was well. His only past medical history was a non-radiological General Practice diagnosis of Osgood-Schlatter disease more than 12 months earlier, symptoms of which had resolved completely.

**Figure 1:**
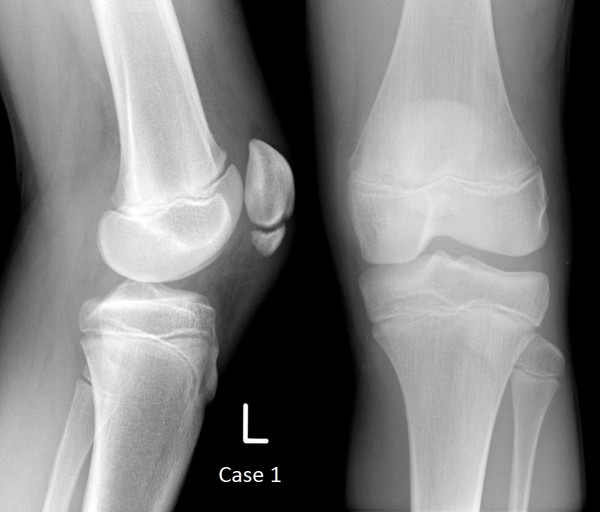
Plain film radiographs of Case 1 at presentation.

**Figure 2:**
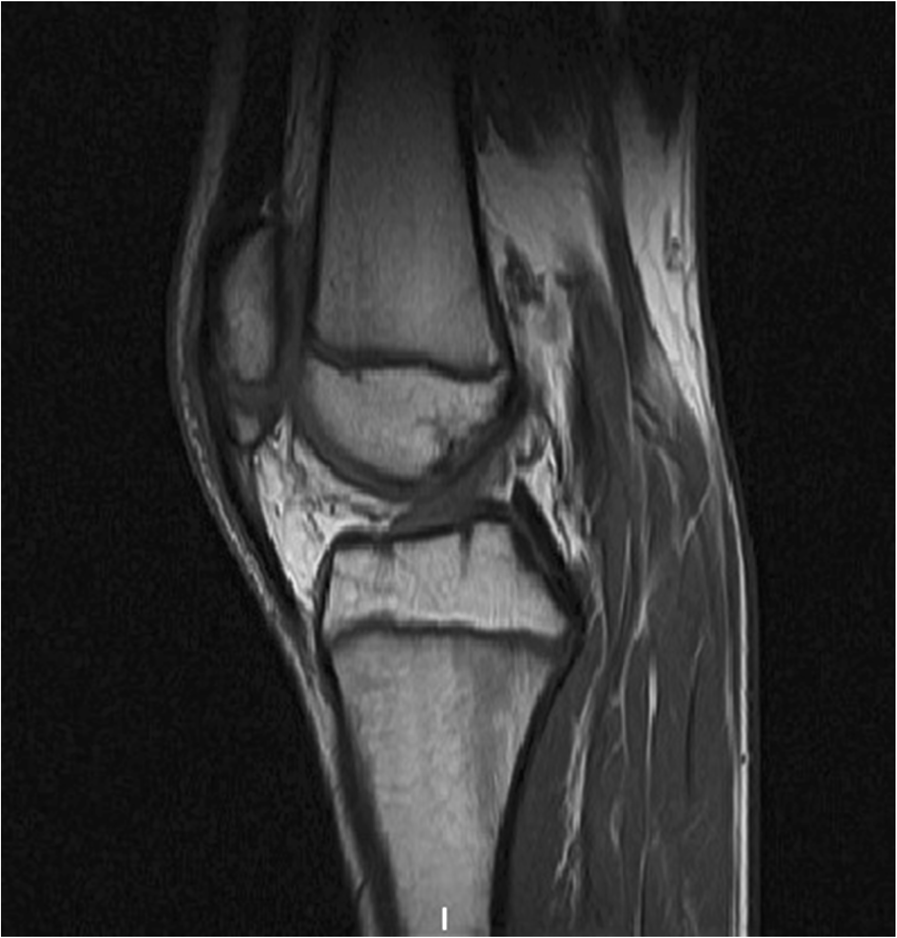
T1-weighted sagittal MRI of Case 1 at 2 weeks.

**Figure 3:**
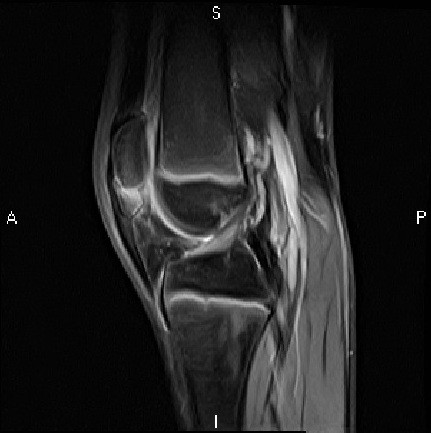
T2-weighted sagittal MRI of Case 1 at 2 weeks.

**Figure 4:**
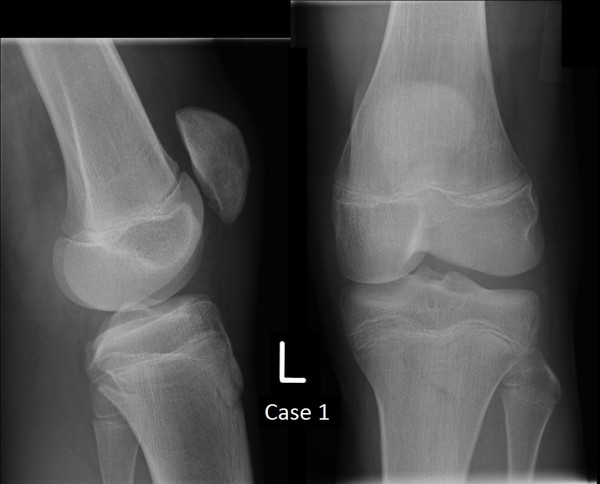
Plain film radiographs of Case 1 at 17 months.

Clinical examination of his knee revealed a small effusion with tenderness at the inferior pole of the patella. He was unable to straight leg raise because of pain. Initial plain film radiography (Figure 1) demonstrated a fracture of the inferior pole of the patella with minimal displacement. Magnetic resonance imaging showed a distinct separation of the inferior pole of the patella (Figure 2) and bone marrow oedema (Figure 3). The knee was immobilised in extension in a brace for a period of 5 weeks, following which he was found to be pain-free. He gradually returned to activity and by 8 weeks had resumed his normal training regime. Plain film radiography at 17 months post injury showed complete union of the patella (Figure 4).

### Case 2

The identical twin of the patient in case 1 presented to the Emergency Department 14 months later, aged 15 years, with an almost identical history. He experienced acute-onset anterior left knee pain whilst taking off for a tumble during a gymnastics training session. He also reported hearing a noise, described as a single “click” or “pop”. His only past medical history prior to this was Osgood-Schlatter disease of the contra-lateral knee diagnosed without radiology three years previously.

**Figure 5:**
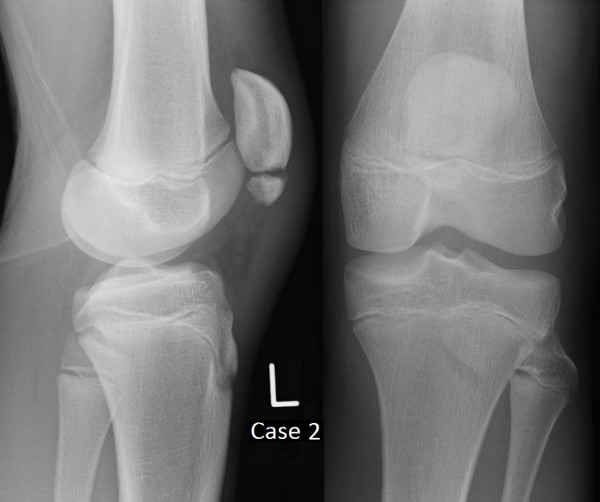
Plain film radiographs of Case 2 at presentation.

Clinical examination of the left knee revealed a small effusion and tenderness at the inferior pole of the patella. He was able to actively straight leg raise but with significant discomfort. Passive flexion of the knee was painful. Initial plain film radiography (Figure 5) demonstrated a fracture of the inferior pole of the patella with minimal displacement.

**Figure 6:**
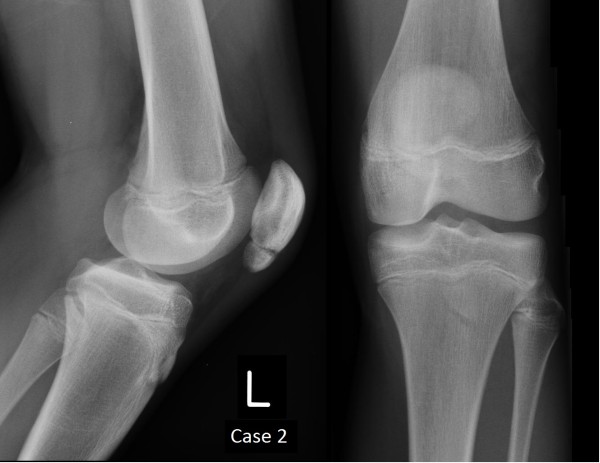
Plain film radiographs of Case 2 at 12 weeks.

His injury was managed in the same manner as his brother’s. The knee was immobilised in extension in a brace for a period of 5 weeks from injury. Pain-free, he resumed activity upon removal of the brace. Plain film radiography at 12 weeks demonstrated evidence of early union (Figure 6). He, also, was able to return to his pre-injury level of training at 8 weeks.

## Discussion

Gymnastics is a hazardous sport [2], especially in the paediatric population, in whom there is a high incidence and severity of injury [3]. One third (33.8%) of gymnastic injuries in children involves the lower extremity [4]. This report describes the presence of a patella fracture in high performing monozygotic twin brothers. To our knowledge no other case of identical patella fractures has been reported in twins.

Significant genetic influence on bone composition has been well documented, heredity and racial factors heavily influencing bone mineral density (BMD) [5]. This is evident in children [6,7] and adults [8-10] alike. Indeed, Friedl et al. attributed seventy percent of the variability in BMD to genetics [11]. Examining twins has further demonstrated genotype to play a major role in the determination of BMD [12]. Monozygotic twins have been shown to have a more highly correlated BMD than dizygotic twins [12,13]. A conclusive explanation for such variation remains elusive with multiple genes shown to be related to bone strength and mass [5]. A lower BMD has been observed in adolescents with fractures [14]. It follows that the genetic composition of these two patients may have placed them at increased risk of suffering a patella fracture. Intrinsic factors, including activity and knee kinematics, at the time of injury would have produced a similar stress pattern in each case. Extrinsic factors such as training intensity, training load, footwear and training surfaces were also very similar in both cases. A recent Australian study of acrobatic gymnastics highlighted the 11–15 years age period specifically as “critical for the occurrence of injury” [15]. Its authors hypothesised that the adolescent growth spurt may be culpable for a greater susceptibility to injury in those undergoing excessive training regimes during this period.

It is difficult to reliably assess the type of fractures sustained. Anatomically identical stress fracture has been described in monozygotic twins previously [16,17]. Chronic stress fracture of the patella is a recognised injury [18], usually preceded by a painful syndrome. No history of anterior knee pain in the months prior to the acute injury was given in these cases. Stress fracture without preceding pain has, however, been reported previously and this diagnosis therefore remains a possibility [19].

The bipartite patella, whilst usually asymptomatic [20,21], is a well documented cause of anterior knee pain, especially following injury [20-23] and unity of bipartite patella has been reported previously [24]. Bipartite patellae are almost always bilateral [24]. Both cases in this report had normal contralateral radiographic appearances. However, without the benefit of pre-morbid radiography, we are unable to exclude this diagnosis.

In the absence of a definitive diagnosis, minimally displaced avulsion or sleeve fracture of the distal pole of the patella was deemed most likely. This type of injury is an uncommon cause of anterior knee pain [25]. There was a distinct separation of the inferior pole from the main body of the patella with a diastasis of less than 3 mm observed. The diagnosis was supported in case 1 following magnetic resonance imaging (Figures 2, 3).

## Conclusion

We believe this is the only published case of identical injury to the patella in monozygotic twins. It corroborates the potential for a genetic influence on susceptibility to fracture and illustrates the need for further research in this area.

### Consent

Written informed consent was obtained from the parent of the patients for publication of this case report and accompanying images. A copy of the written consent is available for review by the Editor-in-Chief of this journal.

## Competing interests

The authors declare that they have no competing interests.

## Authors’ contributions

AJB, GLR, PC. Andrew J Beamish, Gareth L Roberts and Peter Cnudde contributed equally. All authors co-wrote the paper and read and approved the final manuscript.
